# Concordance of poor child feeding and preventive behavior and its predictors in southwest rural Ethiopia

**DOI:** 10.3402/fnr.v60.32207

**Published:** 2016-08-09

**Authors:** Netsanet Fentahun, Carl Lachat, Tefera Belachew

**Affiliations:** 1 Department of Health Education and Behavioral Sciences, College of Health Sciences, Jimma University, Jimma, Ethiopia; 2 Department of Food Safety and Food Quality, Faculty of Bioscience Engineering, Ghent University, Ghent, Belgium; 3 Department of Population and Family Health, College of Health Sciences, Jimma University, Jimma, Ethiopia

**Keywords:** child feeding, preventive behavior, rural Ethiopia

## Abstract

**Background:**

Inappropriate child feeding and caring practices are a major cause of malnutrition. To date, no studies have examined concordance and discordance of child feeding and preventive behavior and their predictors in developing countries.

**Methods:**

We used baseline data generated from A 2-year-longitudinal agriculture-nutrition panel survey conducted from February 9 to April 9, 2014, in nine districts encompassing 20 randomly selected counties in Oromiya Region and Southern Nation, Nationality and Peoples Region in Ethiopia. Households were recruited using the Expanded Program on Immunization sampling method. A total of 623 children under the age of 5 years and their respective caregivers were included in the analyses. Generalized estimating equations were used to account for clustered observations.

**Results:**

Concordance of poor child feeding and preventive behavior was observed in 45.1% of the children, while 45.5% of the children were suffering from discordance of poor child feeding and preventive behavior. Concordance and discordance of poor child feeding and preventive behavior had almost different predictors. Concordance of poor child feeding and preventive behavior was significantly associated with the age of the caretaker of ≥40 years (odds ratio (OR)=2.14; 95% confidence interval (CI): 1.04, 4.41), low household dietary diversity (OR=3.69; 95% CI: 1.93, 7.04), medium household dietary diversity (OR=2.17; 95% CI: 1.17, 4.00), severe household food insecurity (OR=1.72; 95% CI: 1.01, 2.93), and increase with increasing child age.

**Conclusion:**

A substantial number of children in the southwest of rural Ethiopia are exposed to both poor child feeding and preventive behavior. Low household dietary diversity and extreme food insecurity household were predictors of concordance of poor child feeding and poor preventive behavior and provide useful entry points for comprehensive interventions to address child feeding and caring in the area.

Optimal infant and young child feeding can prevent 1.4 million deaths every year (1). As a global public health recommendation, infants should be exclusively breastfed for the first 6 months of life to achieve optimal growth, development, and health (2). Exclusive breastfeeding has a significant effect on the reduction of mortality from diarrhea and pneumonia, the two largest contributors to infant deaths. Continued breastfeeding up to 23 months leads to continued protection against illness, including diarrhea and respiratory infection (3). From 6 months onward, infants enter a particularly vulnerable period of complementary feeding during which they make a gradual transition to eating family foods. The incidence of malnutrition rises sharply during the period from 6 to 18 months of age in most countries, and the deficits acquired at this age are difficult to compensate for later in childhood (4).

Promotion of breastfeeding, oral rehydration therapy, education about complementary feeding, and insecticide-treated materials jointly prevent more than one-third of all deaths (5). Child care practice refers to the provision of time, attention, and support to meet the physical, mental, and social needs of any specific child of any specific household and community (6). It encompasses promoting a safe and healthy environment, provision of adequate health care, psychosocial connections, and emotional support (7). Appropriate child care practice is key to ensure child survival, optimal growth, and development (8).

Malnutrition in Ethiopia is persistently high over the last several years due to poor child feeding and caring practices. In Ethiopia, mothers are responsible for child feeding and caring, while husbands are in control of household assets, purchasing food or provision of financial means to women to purchase food from the market (9). Although the government tries to recognize the role of women and promote active participation in society, the status of women remains poor (10). The Ethiopian government is highly committed to harmonize with a multisectoral approach to addressing malnutrition. The National Nutrition Strategy and the National Nutrition Program are two policy instruments that have been developed to galvanize multisectoral approach (11). Although both documents emphasize both proper child feeding and preventive behavior, there is no evidence regarding to what extent they occur simultaneously and who is affected.

A suitable feeding and use of preventive behavior is needed to identify vulnerable age groups and to monitor interventions in developing countries (12). Most research regarding optimal infant and young child feeding practices, however, have focused on a single component of optimal infant and young child feeding practices at a time. This has complicated communicating research findings, thereby providing poor guidance for policy makers toward the best use of scarce resources. Fragmentation of assessing child health status may have hampered understanding overall feeding patterns and its relation with child health and nutrition outcomes (13). A combination of feeding and use of preventive behavior is key to provide sufficient information on child growth, survival, and nutritional status (14).

The aim of this study was to assess the concordance and discordance of child feeding and preventive behavior and their predictors in southwest rural Ethiopia. These findings are expected to contribute to the formulation of policy and planning, and implementation and evaluation of the nutrition program of the country. It will help to substantiate the arguments for integrated multisectoral interventions to reduce the persistent high level of stunting in the country.

## Methods and materials

### Study design and population

We used baseline data generated from a 2-year-longitudinal panel survey conducted from February 9 to April 9, 2014, in rural communities of south and southwestern Ethiopia encompassing nine districts and 20 counties in Oromiya Region and Southern Nation, Nationality and Peoples Region. A county (Kebele) is the smallest political administrative unit equivalent to a county. Most of the households sampled are situated in Midland (Woina-Dega Zones), and some are in highland (Dega).

This study was a part of Empowering New Generations to Improve Nutrition and Economic Opportunities (ENGINE) project funded by the United States Agency for International Development (USAID) through the Tufts University. ENGINE is a 5-year program aimed to improve the nutritional status of women and young children through sustainable, comprehensive, coordinated, and evidence-based interventions. The sample size of the ENGINE project was 1,200 households. Of the 1,200 households, 623 households had children under the age of 5 years. Therefore, all children under the age of 5 years and their respective caregivers were included in the study.

Nine districts were selected from an ENGINE project area based on logistic feasibility. Lists of all the counties within the nine districts were generated, and two counties were randomly selected in each district. The households were recruited using the expanded program on immunization sampling method (15). First, the county was divided into four quarters with approximately equal household densities. Second, a household in the center of each of the quarters was identified. In each quarter, the enumerators moved in a random direction, determined by spinning a pen, and selected every second house in that direction. The number of households per county was determined with probability proportional to the size of the county. If there were two or more children under 5 years of age in a selected household, only one child was selected randomly.

### Measurements

Data were collected using pre-tested interviewer-administered questionnaire. The project employed a multidisciplinary team comprising 26 data collectors with the background of health and agriculture and six supervisors from one of the two disciplines. A structured pre-tested questionnaire was prepared in Afan Oromo and Amharic and collected using an electronic tablet. Supervisors transferred data to the central database via a wireless internet connection from tablets.

Child feeding and preventive behavior practice indices were created according to previous guidelines (13). A child-feeding index was derived as the summation of breastfeeding, bottle-feeding, and frequency of complementary feeding, child dietary diversity. Similarly, preventive behavior was derived as the sum of washing hands before preparing the food, washing hands before feeding a child, child sleep under a bed net, feeding special foods when a child is ill, and feeding a child special foods after illness. The scores were summed to generate the child feeding and use of preventive behavior indices. Finally, the child feeding and caring indices were dichotomized each as poor ‘1’ for those having a score below median value and good ‘0’ for those having a median value score and above. We defined concordance as children having poor child feeding and poor preventive behavior and discordance as children having poor child feeding and good preventive behavior and good child feeding and poor preventive behavior.

A 24-h qualitative dietary recall was used to measure child dietary diversity score (CDDS). The CDDS was calculated from seven food groups according to the World Health Organization indicators for assessing infant and young child feeding practices 1) Grains/Tubers, 2) Milk, 3) Vitamin A-rich fruits/vegetables, 4) Other fruits/vegetables/juice, 5) Animal protein foods, 6) Eggs, and 7) Legumes (16).

Household dietary diversity score (HDDS) was calculated from 12 food groups according to the Food and Agriculture Organization: 1) Cereals, 2) White, tubers, and roots, 3) Vegetables, 4) Fruits, 5) Meat, 6) Eggs, 7) Fish and other seafood, 8) Legumes, nuts, and seeds, 9) Milk and milk products, 10) Oils and fats, 11) Sweets, and 12) Spices, condiments and beverages. The HDDS ranged from 1 to 12 and ranked from low dietary diversity (≤three food groups), medium dietary diversity (four and five food groups), and high dietary diversity (≥six food groups) (17).

Food insecurity was measured using a Household Food Insecurity Access Scale (HFIAS) adapted from household food insecurity scales that were previously validated for use in developing countries (18). The responses were summed to produce an index of household food insecurity. The distribution of the index of household food insecurity was divided into four categories representing food secure households, occasional food insecurity, moderately food insecure, and severely food insecurity (19).

The influence of cultural norms on child feeding and preventive behavior was assessed using four items with four-point Likert scales (from 1=strongly agree to 4=strongly disagree) adapted from similar studies conducted in low- and-middle-income countries (20). Scale reliability coefficient and experts on the topic were used to check the internal consistency and content validity of the measurements. The internal consistency of the cultural norm scale was 0.9. The distribution of the index of the cultural norm was divided into tertiles representing households that have low adherence to local cultural norms, households that have medium adherence to local cultural norms, and households that have high adherence to local cultural norms on child feeding and caring practices.

The decision-making power of women was measured using 11 items with four-point Likert scales, with 1 representing ‘not at all’ to 4 designating ‘to high extent’, adapted from similar studies conducted in low- and middle-income countries (21). Scale reliability coefficient and experts in the area were used to assess the internal consistency and content validity of the measurements. The internal consistency of the cultural norm was 0.7. The distribution of women's decision-making index divided into tertiles representing low, medium, and high women decision-making power.

Questions were prepared to measure the age of the child, age of the caretaker, sex of the child, caretaker's education, and child health status. Five questions with ‘true/false’ were prepared to measure caretaker's knowledge on nutrition. The distribution of index of nutrition knowledge was ranked and divided into tertiles representing poor, fair, and good nutrition knowledge.

### Data quality

Before data collection, the questionnaire was pre-tested on 5% of the total sample that was not included in the final main sample. The pre-test was conducted in Yem Special District in SNNP Region and Bedele District in Oromiya region, which has similar characteristics to the main sample. A 12-day intensive training was provided to data collectors and supervisors prior to data collection. The training focused on how to ask questions, their meaning, and how to record the answers. The trainees were also encouraged to ask about issues that are unclear, pay close attention, and take careful notes on issues that they are not familiar with. During and after data collection, supervisors monitored the data collection team to ensure their adherence to the study protocol. In addition, the data manager checked all the data submissions from the field on a weekly basis.

### Data analysis

The data were checked for distribution, missing values, and outliers, cleaned, and analyzed using Statistical Package for Social Sciences (SPSS) version 20.0. (Armonk, NY: IBM Corp) and STATA 11 Software (Stata Corp, College Station, TX). Descriptive analyses were conducted to describe the characteristics of the study participants. Since the study was conducted in different districts, we accounted for the clustered nature of the measurements during the analyses. For this purpose, generalized estimating equations were used to adjust the standard errors. A generalized estimating equation with a logic function was used to determine the predictor of concordance and discordance of child feeding and caring practices in a child.

An exchangeable correlation structure was chosen for the main models by assuming two observations are equally correlated within a cluster, with no correlation between observations from different cluster correlations. The generalized estimating equations were adjusted for decision-making power of women, the age of caretaker, educational status of caretaker, the age of child, household dietary diversity, the influence cultural norms on child feeding and preventive behavior, and household food insecurity. Multicollinearity and interaction terms were checked during generalized estimating equations analyses. Finally, the results were reported as odds ratio (OR) and 95% confidence intervals (CI).

### Ethical considerations

Ethical approval was obtained from the Institutional Review Board of the College of Health Sciences of Jimma University, Ethiopia, and the Institutional Review Board of Tufts University, USA. Written permission was obtained from each responsible body and informed verbal consent was obtained from each study participant. We received a waiver of documentation of informed consent from the Institutional Review Boards. The letter stated that respondents do not need to sign the consent statement because many are illiterate. However, there was a place on the form for the enumerator to sign in order to indicate that participants have read the consent form and that the person had agreed to participate. Data were registered and stored anonymously, and the questionnaire was administered in a confidential way.

## Results

A total of 623 children under the age of 5 years were involved in the study. Out of 623, 13 children with missing data on child feeding and preventive behavior were excluded from the analyses. Table 1 illustrates the child feeding practices in southwest rural Ethiopia. Fifty-nine percent of mothers exclusively breastfed their index children for 6 months. The most common reasons cited for terminating breastfeeding were pregnancy (33.9%), not having enough breast milk (16.1%), and being tired of breastfeeding (12.6%). Seventy-one percent of mothers started complementary feeding at the age of 6–9 months. The median age of the introduction of complementary feeding was 6 months, and 43.8 and 59.0% of mothers did not administer special food to their child during and after illness, respectively.

**Table 1:** Child feeding practices in southwest rural Ethiopia, 2014

		Frequency (*n*)	Percentage
Did you ever breastfeed?	No	120	19.7
	Yes	490	80.3
How long after birth did you first put to the breast?	Within first hour after birth	262	53.5
	After first hour	181	36.9
	After 1 day	39	8.0
	Don't remember	8	1.6
During the first 3 days after delivery, did you give the liquid	No	202	41.2
that came from your breasts to your baby?	Yes	283	57.8
	Don't know	5	1.0
During the first 3 days after delivery, did you give your baby anything	No	450	91.8
else to eat or drink before feeding him/her breast milk?	Yes	38	7.8
	Don't know	2	0.4
What did the baby eat or drink before feeding him/her breast milk?	Milk (other than breast milk)	2	5.7
	Butter	14	40.0
	Plain water	7	20.0
	Water with sugar or salt	11	31.4
	Traditional herbs with water	1	2.9
Duration of breast feeding	<6 months	41	23.8
	6–8 months	2	1.2
	9–12 months	5	2.9
	13–24 months	124	72.1
Did the baby drink anything from a bottle with a nipple	No	66	10.8
yesterday or last night?	Yes	544	89.2

Out of 592 children, 91.0% had either feeding or preventive behavior problems. Of those children, 45.1% had concordance of poor child feeding and preventive behavior. An estimated 32.3% of children had only poor child feeding and good preventive behavior, and 13.2% of children had poor preventive behavior and good child feeding practices. Only 9.4% of children, however, had both good child feeding and preventive behavior.

We also observed that out of 610 children, 85.1% did not sleep under a bed net during the night before the interview, and 23.6% reported an illness in the past 2 weeks. Out of 65 children who had diarrhea, 58.5% were not given any liquid. Similarly, of 136 children who had any illness or symptoms, 51.5% reported to have sought health services outside the home. Regarding health seeking behaviors, 45.7% received help from private clinics, while the health extension workers treated 28.6% children.

Table 2 describes the bivariate associations of concordance of poor child feeding and preventive behavior in southwest rural Ethiopia. Age of the child, household dietary diversity, household food insecurity, educational status of the caretaker, and age of the caretaker had significant association with concordance and discordance of poor child feeding and preventive behavior. However, the influence of cultural norms on child feeding caring practice was only associated with poor child feeding and good preventive behavior.

**Table 2:** Bivariate association of concordance and discordance of child feeding and preventive behavior in southwest rural Ethiopia, 2014

	Concordance (poor child feeding and poor preventive behavior)	Discordance (poor child feeding and good preventive behavior)	Discordance (good child feeding and poor preventive behavior)	*p*
Age of the child				0.001*
6–8 months	3 (0.5%)	11 (1.9%)	2 (0.3%)	
9–12 months	12 (2.0%)	26 (4.4%)	10 (1.7%)	
13–36 months	155 (26.2%)	31 (5.2%)	133 (22.5%)	
37–60 months	97 (16.4%)	10 (1.7%)	46 (7.8%)	
Total	267 (45.1%)	78 (13.2%)	191 (32.3%)	
Educational status of the caretaker				0.05**
Illiterate	186 (31.4%)	43 (7.3%)	122 (20.6%)	
Primary education	79 (13.3%)	33 (5.6%)	64 (10.8%)	
Secondary education	2 (0.3%)	2 (0.3%)	5 (0.8%)	
Total	267 (45.1%)	78 (13.2%)	191 (32.3%)	
Age of the caretaker				0.001**
≤20 years	12 (2.0%)	10 (1.7%)	20 (3.4%)	
21–30 years	134 (22.6%)	35 (5.9%)	108 (18.2%)	
31–40 years	90 (15.2%)	26 (4.4%)	55 (9.3%)	
>40 years	31 (5.2%)	7 (1.2%)	8 (1.4%)	
Total	267 (45.1%)	78 (13.2%)	191 (32.3%)	
Household dietary diversity				0.001*
Low	141 (23.8%)	9 (1.5%)	84 (14.2%)	
Medium	110 (18.6%)	55 (9.3%)	78 (13.2%)	
High	16 (2.7%)	14 (2.4%)	29 (4.9%)	
Total	267 (45.1%)	78 (13.2%)	191 (32.3%)	
Cultural norms				0.05***
High	90 (16.2%)	14 (2.5%)	72 (13.0%)	
Medium	89 (16.1%)	30 (5.4%)	50 (9.0%)	
Low	70 (12.6%)	27 (4.9%)	56 (10.1%)	
Total	249 (44.9%)	71 (12.8%)	178 (32.1%)	
Household food insecurity				0.001*
Food secure	88 (14.9%)	46 (7.8%)	76 (12.8%)	
Occasional	14 (2.4%)	4 (0.7%)	25 (4.2%)	
Moderate	80 (13.5%)	11 (1.9%)	56 (9.5%)	
Sever food insecurity	85 (14.4%)	17 (2.9%)	34 (5.7%)	
Total	267 (45.1%)	78 (13.2%)	191 (32.3%)	

* Significant for all

** significant for both poor child feeding and caring practices

*** significant for both good feeding and poor caring practice.

Table 3 shows the comparison of factors associated with concordance and discordance of poor child feeding and preventive behavior in southwest rural Ethiopia. Concordance and discordance of poor child feeding and preventive behavior had almost different predictors. Children at an early age, having a young caretaker and living in high household dietary diversity were less likely to suffer from concordance of poor child feeding and preventive behavior. Children who live in a food insecurity household suffered from both concordance and discordance of poor child feeding and preventive behavior. Children who live in a household with a high adherence to local cultural norms only suffered from discordance of poor child feeding and preventive behavior.

**Table 3:** Comparison of factors associated with concordance and discordance of child feeding and preventive behavior in southwest rural Ethiopia, 2014

	Concordance (poor child feeding and poor preventive behavior)	Discordance (poor child feeding and good preventive behavior)	Discordance (good child feeding and poor preventive behavior)
	
	AOR (95.0% CI)	AOR (95.0% CI)	AOR (95.0% CI)
Age of the caretaker			
≤20 years	0.5 (0.26, 1.11)	1.3 (0.66, 2.53)	1.3 (0.52, 3.33)
31–40 years	1.2 (0.80, 1.84)	0.8 (0.51, 1.22)	1.5 (0.76, 2.87)
≥40 years	2.1 (1.04, 4.41)*	0.4 (0.17, 0.94)*	1.7 (0.58, 4.96)
21–30 years (Ref)	1	1	1
Age of the child			
6–8 months	0.1 (0.03, 0.42)**	0.2 (0.03, 0.72)**	10.5 (3.30, 33.47)**
9–12 months	0.2 (0.07, 0.34)**	0.4 (0.19, 0.97)*	8.9 (3.33, 23.56)**
13–36 months	0.6 (0.39, 0.92)**	1.5 (0.98, 2.41)	2.0 (0.83, 4.62)
37–60 months (Ref)	1	1	1
Educational status of the caretaker			
Illiterate	2.5 (0.55, 11.23)	0.9 (0.24, 3.09)	0.4 (0.07, 2.21)
Primary education	2.1 (0.47, 9.72)	0.9 (0.24, 3.12)	0.7 (0.12, 3.72)
Secondary education (Ref)	1	1	1
Household food insecurity			
Severe	1.7 (1.01, 2.93)*	0.6 (0.34, 1.08)	1.4 (0.66, 2.95)
Moderate	1.1 (0.67, 1.76)	1.2 (0.71, 1.94)	0.6 (0.28, 1.43)
Occasionally	0.5 (0.24, 1.03)	2.8 (1.40, 5.60)**	0.7 (0.22, 2.34)
Food secure (Ref)	1	1	1
Household dietary diversity			
Low	3.7 (1.93, 7.04)**	0.9 (0.05, 1.72)	0.2 (0.07, 0.58)*
Medium	2.2 (1.17, 4.00)*	0.8 (0.42, 1.34)	1.3 (0.62, 2.74)
High (Ref)	1	1	1
Women decision-making			
Low	0.8 (0.48, 1.25)	1.2 (0.72, 1.92)	1.3 (0.66, 2.69)
Medium	0.8 (0.48, 1.18)	1.2 (0.72, 1.85)	1.1 (0.56, 2.34)
High (Ref)	1	1	1
Nutrition knowledge of the caretaker			
Poor	1.3 (0.79, 2.19)	0.8 (0.47, 1.38)	0.9 (0.37, 2.05)
Fair	1.0 (0.64, 1.60)	1.2 (0.72, 1.85)	1.2 (0.59, 2.29)
Good (Ref)	1	1	1
Cultural norms			
High	1.0 (0.55, 1.66)	1.3 (0.75, 2.26)	0.4 (0.19, 0.94)*
Medium	1.0 (0.59, 1.63)	1.0 (0.61, 1.72)	1.1 (0.54, 2.15)
Low (Ref)	1	1	1

AOR: adjusted odds ratio, CI: confidence interval, (Ref): reference category.

* Significant at *p<*0.05

** significant at *p<*0.001.

## Discussion

This study presents findings on the concordance and discordance of child feeding and preventive behavior and compares factors associated with child feeding and preventive behavior in southwest of rural Ethiopia. Almost all children were suffering from either child feeding or preventive behavior problems. Of those children and in almost half of the children in the sample, poor child feeding and preventive behavior occurred. In only a minority of children, however, relatively good feeding practices were observed despite poor preventive behavior. The presence of low health services, poor sanitation, and lack of commitment addressing child nutrition in developing countries may have contributed to the problem of low feeding and preventive behavior. Therefore, a reduction in child mortality can only be reached when child feeding and preventive behavior are prioritized in national policies and strategies (3, 22).

Gender-based differences in concordance and discordance of poor child feeding and poor preventive behavior were not observed in our sample. The reasons were cross-sectional nature of the data and cultural influence to tell the gender-based difference in child feeding and preventive behavior. This result was supported by a study conducted in rural Cambodia (14), which indicated that promoting gender equality in child feeding and preventive behavior has a positive impact on the reduction of childhood malnutrition and mortality. Studies from Bangladesh and India, however, showed that male children had higher child feeding and caring practices (18, 23).

Caretaker education was not significantly associated with concordance and discordance of poor child feeding and preventive behavior in our sample. The reasons were that the majority of the caretakers were homogenous in their educational status and they are illiterate and 1–12 grade education curricula had no nutrition education components. Therefore, education alone is not the guarantee for best child feeding unless the caretaker is equipped with nutrition knowledge and skills. This finding is supported by data from rural Cambodia (10), but findings from rural Bangladesh showed that maternal education was significantly associated with child feeding and caring practices (18). A similar study conducted in Benin showed that maternal education through 4 years of schooling was associated with improved child feeding. However, maternal schooling beyond 4 years is negatively associated with child weight (24). Findings from the United States show that college-educated mothers were significantly more likely to comply with supplied feeding recommendations (25).

Children not living in households with high household dietary diversity were more likely to suffer from concordance of poor child feeding and poor preventive behavior but not in discordance. Inappropriate complementary feeding practices increase the incidence of malnutrition, a high rate of infectious diseases, and adversely affect child growth and development (2, 26). Demographic and health surveys data of 11 countries showed that household dietary diversity was associated with child feeding and preventive behavior (27), indicating household dietary diversity to be a vital component of ensuring child feeding and preventive behavior.

Children living in severe food insecurity households were more likely to suffer from concordance of poor child feeding and poor preventive behavior but not in discordance. As observed in Bangladesh, children from households suffering from occasionally or intermittently household food insecurity were more likely to have high caring practices (18). Facing household food insecurity, women are particularly vulnerable to nutrient inadequacies related to physiological vulnerability during childbearing and experience fatigue, which limits their ability to fully satisfy infant needs. The limitations impair child growth and cognitive development, which may persist into adulthood and transmit to the next generation (28) indicating that household food security is the vital component of assuring child feeding and preventive behavior. Therefore, household food insecurity problems must be addressed to reduce malnutrition in southwest rural Ethiopia.

Women decision-making power was not associated with concordance and discordance of poor child feeding and preventive behavior in our sample. A study conducted in rural Chad showed that decision-making power of women was associated with child feeding and preventive behavior (29). Caregivers have great influence on child-feeding practices, willingness to seek advice during childhood illnesses, and the number of individuals available to assist with domestic tasks was a factor associated with child feeding and nutritional status (30). This study focused on the decision-making power of women on agricultural activities. Increasing decision-making power of women on agricultural activities alone had an effect on child feeding and preventive behavior. Therefore, women decision-making on agriculture and nutrition must be promoted to improve child feeding and preventive behavior.

At younger ages, children (6–12 months) were less likely to suffer from concordance and discordance of poor child feeding and preventive behavior, but they suffer more from discordance of poor child feeding and preventive behavior. The empirical evidence shows that child feeding and preventive behavior are interdependent (31). A similar study conducted in rural Cambodia and Ghana showed that the age of the child was strongly associated with child feeding and preventive behavior (14, 32), indicating that concordance of child feeding and preventive behavior with the age of the child is a vital issue to address any child feeding and preventive behavior problems.

The nutrition knowledge of the caretaker was not associated with concordance and discordance of poor child feeding and preventive behavior in our sample. Due to the homogeneous nature of the study participants, nutrition knowledge of caretaker was not significant for child feeding and caring practices. The study conducted in Ethiopia showed that nutrition knowledge has a positive effect on child feeding and preventive behavior (33). A similar study conducted in Ghana revealed that nutrition knowledge of the mother was positively associated with child feeding practices (31). Even though nutrition knowledge of caretaker was not associated with low child feeding or with low caring, creating awareness about child feeding and preventive behavior is the best option to prevent malnutrition, morbidity, and mortality in southwest rural Ethiopia.

Discordant poor child feeding and preventive behavior was found in children living in households with a high adherence to local cultural norms. In low- and middle-income countries, acceptable infant feeding practices are complex and vary from one society to another due to sociocultural factors (4). A study conducted in Ghana and Ethiopia (20, 34) reported that there are many sociocultural factors and misconceptions that are associated with child feeding and preventive behavior. Therefore, nutrition education that considers the sociocultural factors and misconceptions is essential to effectively address child feeding and use of preventive behavior in low- and middle-income countries.

The strength of the study was its focus on concordance and discordance of poor child feeding and preventive behavior in southwest of rural Ethiopia. Estimates on the concordance and discordance of poor child feeding and preventive behavior in children can guide planning, implementation, and evaluation of integrated promotion of optimal caring and feeding behaviors in low- and middle-income countries. Such knowledge can also strengthen partnerships between nutrition, agriculture, and health by clearly indicating specific tasks among various sectors that work on reduction of chronic malnutrition. However, since the study uses the data from the post-harvesting season, it may not be generalized to other seasons. In addition, due to the cross-sectional nature of the data, causal effects cannot be inferred.

## Conclusion

Almost half of the children suffered from concordance of poor child feeding and preventive behavior in rural southwest Ethiopia. Household dietary diversity, household food insecurity, age of the child, and age of the caretaker were associated factors of concordance of poor child feeding and preventive behavior in southwest rural Ethiopia. This finding provides a useful entry point to address child feeding and preventive behavior in an integrated way through multisectoral collaboration. Specific attention is required for children during the complementary feeding period and those in the food insecure households who are highly at risk. The findings call for comprehensive interventions to address child feeding and preventive behavior in the area to prevent the pervasively high level of stunting in rural Ethiopia.
